# Lack of evidence for HEV infection in Baltic sea mussels (Mytilidae)

**DOI:** 10.1186/s12879-025-10727-7

**Published:** 2025-03-13

**Authors:** M. Rastar-Tangeten, M. Mader, S. Schmiedel, S. Weidemann, S. Chalissery, J. Kempski, T. Staufenberger, O. Mazaheri, J. M. Brandner, M. M. Addo, C. Ackermann, A. Wolski, S. Reucher, J. Wenzel, M. Lütgehetmann, M. Schemmerer, J. Schulze Zur Wiesch, S. Pischke

**Affiliations:** 1 Department of Medicine, University Medical Center Hamburg-Eppendorf, Hamburg, Germany; 2 German Center for Infection Research (DZIF), Hamburg-Lübeck-Borstel-Riems Partner Site, Hamburg, Germany; 3 Institute of Pathology, University Medical Center Hamburg-Eppendorf, Hamburg, Germany; 4 Kieler Meeresfarm GMBH CO KG, Kiel, Germany; 5 Geschäftsbereich Sicherheit & Compliance, University Medical Center Hamburg-Eppendorf, Hamburg, Germany; 6 Institute of Medical Microbiology, Virology and Hygiene, University Medical Center Hamburg-Eppendorf, Hamburg, Germany; 7 National Consultant Laboratory for HAV and HEV, Institute of Clinical Microbiology and Hygiene, University Medical Center Regensburg, Regensburg, Germany

**Keywords:** Hepatitis E, Mussels, HEV, Shellfish, Mytilidae

## Abstract

**Background and aims:**

Hepatitis E virus (HEV), similar to hepatitis A virus (HAV), has been linked to cases associated with mussel consumption, and several studies have detected HEV in commercially available mussels. While extensive data exists on HEV contamination in mussels from tropical regions, the Mediterranean Sea, and the North Sea, there is a lack of information regarding the potential risk posed by common mussels (*Mytilidae*) from the Baltic Sea. Furthermore, no experimental studies have investigated the ability of Baltic Sea mussels to be infected with HEV.

**Material and methods:**

Healthcare workers (*n* = 447) from the University Medical Center Hamburg Eppendorf, Germany, were surveyed regarding their mussel consumption habits and tested for anti-HEV IgG using a commercially available assay. Commercially sourced Baltic Sea *Mytilidae*, obtained from local retailers, were tested for HEV using a validated PCR method. Additionally, 50 live *Mytilidae* were experimentally spiked with HEV, followed by dissection and separate PCR analysis of the gastrointestinal tract, gonads, and muscle tissue.

**Results:**

There was no significant difference in the likelihood of anti-HEV IgG positivity between individuals who frequently consumed mussels and those who did not. None of the 40 commercial mussel samples tested were positive for HEV. HEV RNA was detected in the gastrointestinal tract of experimentally exposed Mytilidae specimen but not in their gonads or muscle tissue. It was observed that HEV RNA persisted in the gastrointestinal tract for more than 14 days but not beyond 21 days.

**Discussion:**

This study provides no evidence of HEV contamination in commercially sourced Baltic Sea mussels, as all tested samples were negative for HEV RNA. Moreover, no significant association was observed between mussel consumption and anti-HEV IgG positivity among healthcare workers. Experimental exposure revealed that HEV RNA can persist in the gastrointestinal tract of Baltic Sea mussels for over 16 days but less than 24 days, while no viral presence was detected in the gonad or muscle tissue. These findings suggest a minimal risk of HEV transmission through mussels from the Baltic Sea but highlight the need for further studies to understand their role in HEV ecology.

## Introduction

Hepatitis E virus (HEV) is a single-stranded RNA virus classified as Paslahepevirus balayani within the Hepeviridae family (International Committee on Taxonomy of Viruses: ICTV). In Europe, HEV genotype 3 is a zoonotic pathogen, primarily transmitted via undercooked pork, though shellfish have also been implicated as a potential source of infection, akin to hepatitis A virus (HAV). For instance, a 2009 cruise ship outbreak of HEV was linked to mussel consumption [1].

Shellfish, a broad category including mussels, snails, and crustaceans, are often exposed to viruses via contaminated coastal waters. Studies from European coastal regions have yielded varying results on the prevalence of HEV in mussels. While HEV RNA was not detected in mussels from Denmark or France [2, 3], other studies reported high prevalence rates, such as 85.4% in mussels from Scotland’s North Sea coast and 24.4% in Spanish mussels [4, 5]. HEV RNA persistence and bioaccumulation experiments suggest variability in virus retention among shellfish species. Beyond Europe, HEV contamination has also been observed in mussels and clams from Japan and China, with genotype 4 HEV identified in Asia [6, 7].

Handling raw seafood may also present a significant exposure risk, as demonstrated by a study of seafood processing workers in China, where anti-HEV IgG prevalence was higher in individuals with direct contact with raw seafood [8]. Together, these findings underscore the potential role of shellfish as a vector for HEV transmission.

Despite these insights, the risk of HEV transmission via common mussels (*Mytilidae*) from the Baltic Sea remains unexplored. In Germany, Baltic Sea mussels are a popular local delicacy, but critical questions remain unanswered: How long can HEV persist in mussels from the Baltic Sea? Can the virus replicate within mussel tissues? Does this pose a risk for human infection? To address these gaps, we analyzed commercially sourced Baltic Sea mussels, conducted experimental HEV exposure studies, and compared anti-HEV seroprevalence among individuals with differing mussel consumption habits.

## Materials and methods

### Regulations

As the testing of hospital employees was anonymous and utilized residual material from routine blood samples, no formal ethics vote was required according to local regulations and Hamburg hospital law. However, the study was approved by the Hamburg Medical Association Ethics Committee (approval number PV7049) for the human component, including the questionnaire and testing. All participants provided informed consent before completing the questionnaire.

The study was also approved for safety by the Department of the Interior of the City of Hamburg (correspondence dated 27 August 2021).

Regarding the use of mussels, formal approval from an Animal Ethics Committee was not required, as mussels are invertebrates. It is generally assumed that invertebrates, such as mussels, lack consciousness, and therefore, no formal ethical review was necessary for their use in this study.

### Evaluation of anti-HEV IgG positivity associated with mussel consumption

To evaluate the association between mussel consumption and anti-HEV IgG positivity, 447 employees of the University Medical Center Hamburg-Eppendorf were surveyed. A small subgroup of 10 subjects did not reply to this question and were thus not included in the analysis, leading to 437 analyzable questionnaires. Participants were asked how often they consumed cooked or fried seafood, raw shellfish (e.g., oysters), and raw fish (e.g., sushi) per year. Responses were categorized as “never,” “1–5 times per year,” “6–12 times per year,” or “more than 12 times per year.” This newly developed survey was conducted anonymously, with each questionnaire assigned a unique code linked to the corresponding serum sample for anti-HEV IgG testing. Testing was performed using the Wantai ELISA kit (Wantai, Beijing, China). The design ensured that individual results could not be retrospectively identified.

### PCR testing of commercial mussels

Forty commercially sold *Mytilidae* mussels were purchased and tested for HEV RNA within 48 h of purchase. While the precise catch location was unavailable, the mussels carried MSC (Marine Stewardship Council) certification. Samples were stored at 4 °C until testing using the “cobas HEV PCR” kit and the fully automated Roche Cobas 6800 system (Roche, Mannheim, Germany). The assay followed the manufacturer’s instructions and was calibrated using the first International Standards for hepatitis E virus RNA for nucleic acid amplification technique (NAT)-based assays, ensuring reliable quantification and interlaboratory consistency.

### Experimental HEV exposure of mussels

Fifty live *Mytilidae* mussels from the Baltic Sea were placed in a 40-liter aquarium containing 35 L of natural Baltic Sea water at room temperature. After confirming the absence of HEV RNA in water and mussels on day 3, highly concentrated HEV (genotypes 1 and 3; enveloped and non-enveloped particles) was added on day 4 (Table 1) to achieve a final concentration of 1.13 × 10² copies/mL. This dosage ensured that each mussel filtered over 3.5 million HEV RNA copies within 24 h.

**Table 1:** Calculation of HEV concentration in input sample volume (all samples have been added simultaneously)

HEV Source Sample No.	Origin	Input sample conc. [c/ml]	Input sample Volume [ml]	Output sample Volume [ml]	Output sample conc. [c/ml aquarium water]
1	human faeces (genotype 3)	6.1 × 10^5^	0.500	35,000	**8.8 × 10** ^**0**^
2	Faeces from experimentally infected chimeric mice (genotype 1), proven infectivity	1.0 × 10^5^	1.750	35,000	**5.0 × 10** ^**1**^
3	human faeces (genotype 3)	3.1 × 10^6^	0.568	35,000	**5.0 × 10** ^**1**^
4	human plasma (genotype 3)	3.1 × 10^5^	0.550	35,000	**4.9 × 10** ^**0**^

Aeration was maintained using air pumps, and mussels were fed weekly with approximately 1 g of yeast. To minimize external contamination, the aquarium was sterilized with UV irradiation between water changes. On day 8, the water was replaced with fresh seawater, and subsequent PCR testing was monitored for any increase in HEV RNA, which would indicate viral replication in the mussels.

### Harvesting, dissection, and PCR testing of exposed mussels

**Fig. 1:**
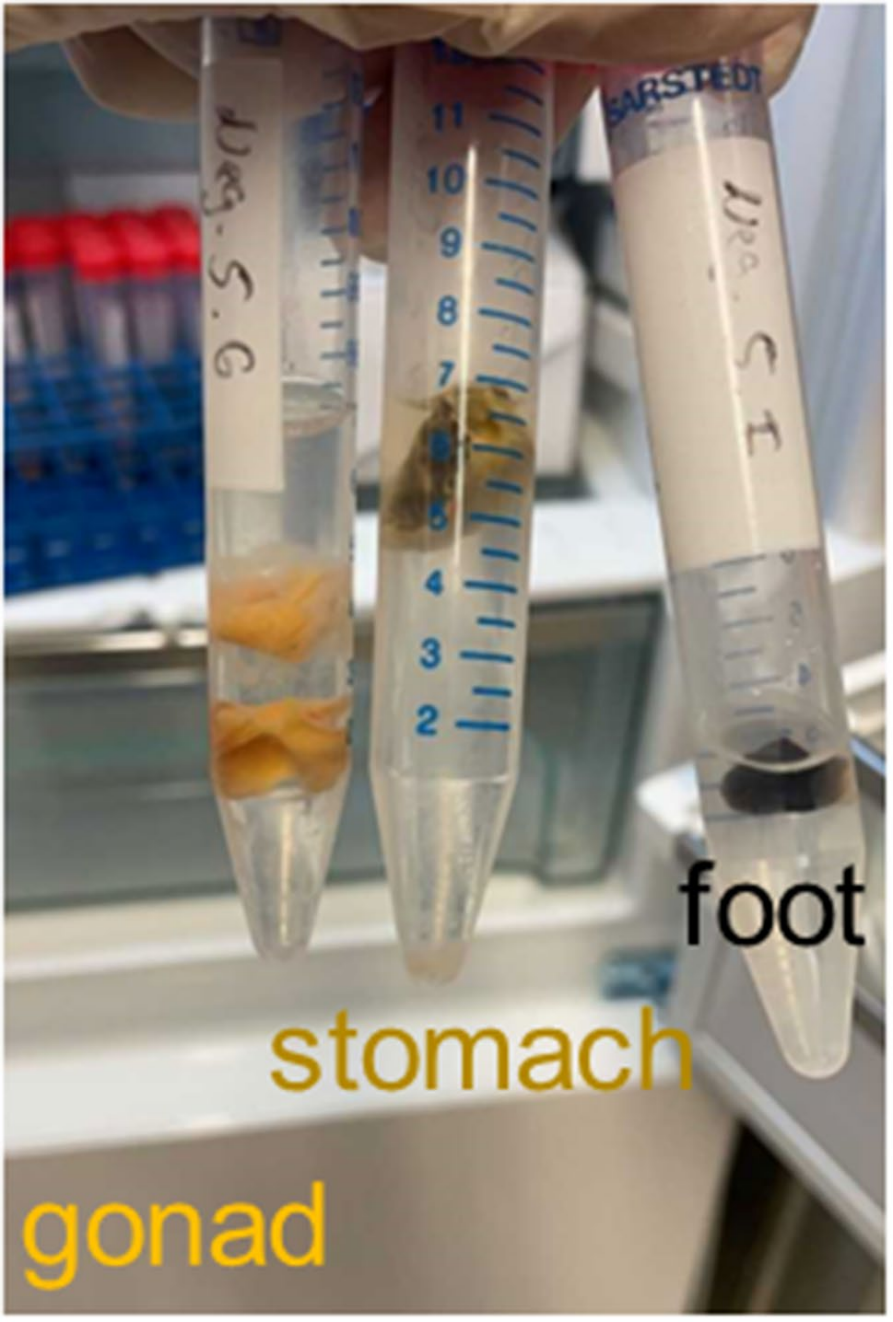
Dissection of mussels in stomach, gonad, and foot (muscle), before separate PCR testing

Mussels were harvested in five stages: day 10 (*n* = 10), day 17 (*n* = 6), day 24 (*n* = 6), day 38 (*n* = 6), and day 43 (*n* = 6). Each mussel was dissected (Fig. 1) and all tissue samples were lysed (Precellys 24, Bertin, Rockville, US) using 2 ml tubes prefilled with ceramic beads (Precellys Lysing Kit) and 1 ml RNA and DNA free PCR grade water, and three tissues (digestive tract, gonad, and muscle) were separately tested for HEV RNA. The proof of concept data was generated by the diagnostic grade IVD assay using the Roche cobas system. Due to diagnostic work overload, this system was unavailable for a larger set of mussels and multiple time points for the follow-up experiments. Therefore the commercial Altona Diagnostics RealStar HEV PCR Kit 2.0 (Altona Diagnostics, Hamburg, Germany) using a similar PCR target region was used and performed in the research laboratory, according to the manufacturer’s protocol.

Due to environmental degradation and turbidity, seawater was replaced with tap water mixed with 2.2% sea salt on day 38. The aquarium underwent UV sterilization before water replacement. On day 50, all mussels had perished, and the experiment concluded. Mussel stool samples collected from the aquarium floor were also tested for HEV RNA.

### Quality control and PCR analysis

The Roche Cobas 6800 was used for initial HEV RNA testing, while the Altona Diagnostics PCR kit (Altona Diagnostics, Hamburg, Germany) was utilized for subsequent analyses. The primers and HEV-specific probe (JVHEVF 5′GGTGGTTTCTGGGGTGAC; JVHEVR 5′AGGGGTTGGTTGGATGAA, TaqMan minor groove binding probe JVHEVPh 5′FAM-TGATTCTCAGCCCTTCGC-MGB) have been described previously [9]. The temperature profile and Utility Channel software settings for the Cobas included a predefined uracil-DNA N-glycosylase incubation step, a pre-PCR step (1 cycle: 55 °C for 120 s, 60 °C for 360 s, 65 °C for 240 s), 5 cycles of 95 °C for 5 s and 55 °C for 30 s (1st measurement at the end of each cycle) followed by 45 cycles of 91 °C for 5 s and 58 °C for 25 s (2nd measurement at the end of each cycle) and finished with a predefined cooling step.

The Roche Cobas Omni channel chemistry uses a variant of the Hawk Z05 polymerase that uses Mg2 + and can also perform the RT step. This eliminates the need for the classic 95’C activation/inactivation step.

The HEV concentration of input samples was verified with the RealStar HEV PCR-Kit 2.0. These assays were calibrated to the WHO HEV standard for consistency and accuracy across testing methods.

For feasibility reasons, the previously described Cobas 6800 was not available for all tests; consequently, these PCR tests were performed with the Altona Diagnostics PCR Kit. The concentration of each source HEV input sample was measured by the Realstar HEV PCR-Kit 2.0 (Altona Diagnostics, Hamburg, Germany; extraction by Quiakit, Qiagen, Germany).

**Figure:**
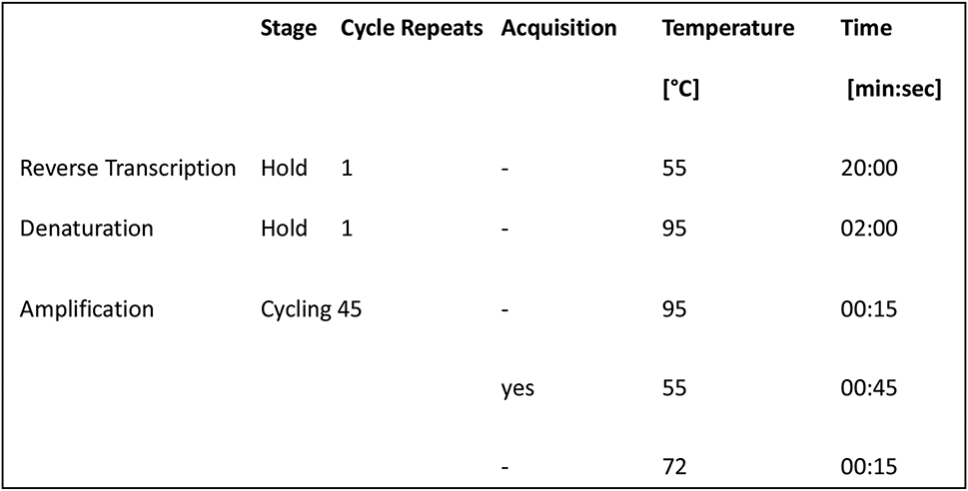

PCR Temperature Profile and Dye Acquisition, Altona Diagnostics PCR:

The procedures for nucleic acid extraction and polymerase chain reaction (PCR) were conducted per the manufacturers’ instructions.

## Results

**Fig. 2:**
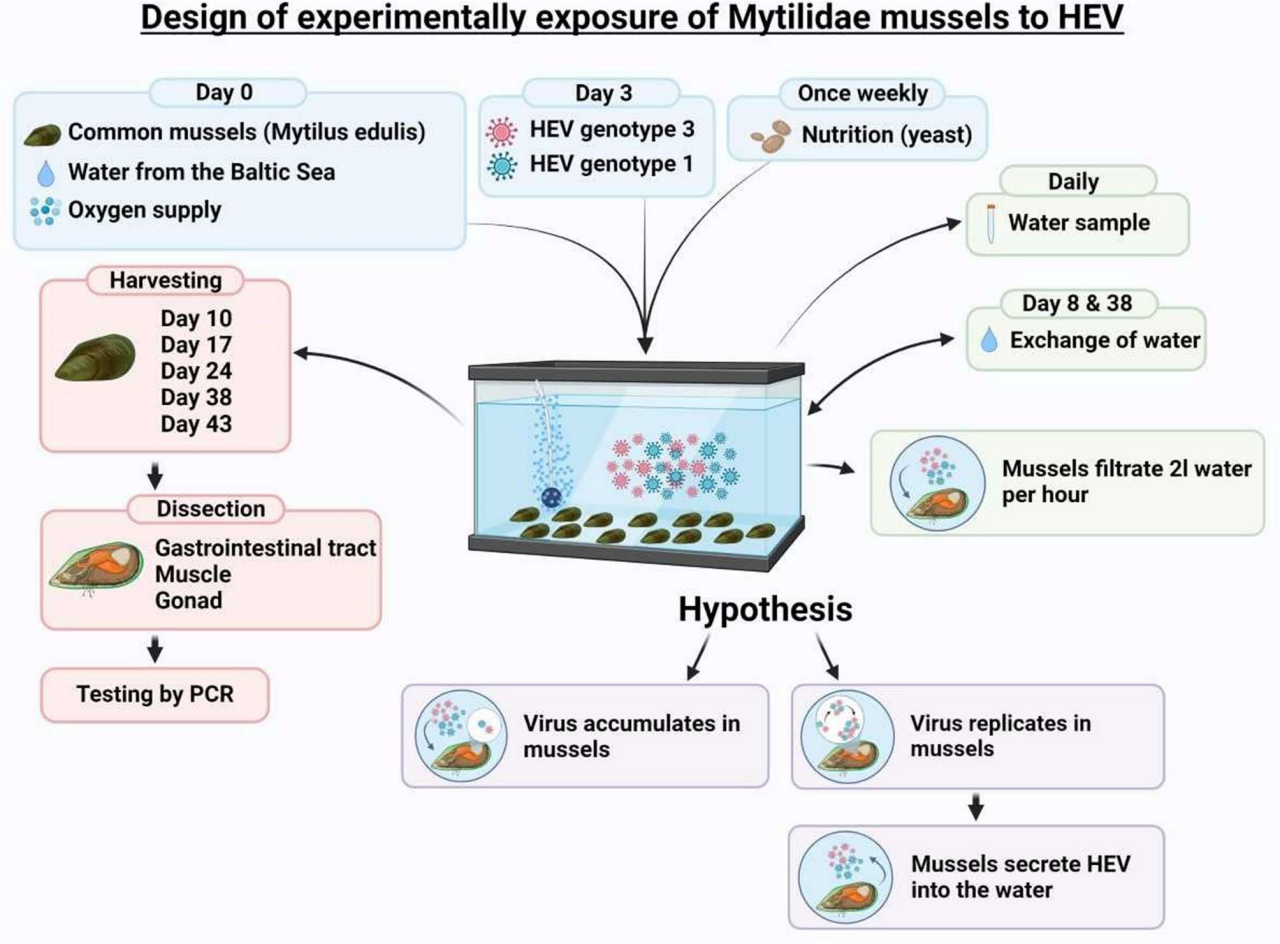
Experimentally exposure of Mussels to HEV

To evaluate the risk of HEV transmission through Baltic Sea *Mytilidae*, three investigative approaches were employed:

1. Anti-HEV IgG seroprevalence concerning mussel consumption:
  A cohort of 447 healthcare workers at the University Medical Center Hamburg-Eppendorf was assessed for anti-HEV IgG seroprevalence and surveyed about their dietary habits.
  The question of whether and how often they eat mussels was answered by 437/447 probands. 190/437 individuals (43%) never ate cooked mussels, while 247/437 (57%) occasionally consumed them. The anti-HEV IgG seroprevalence did not significantly differ between non-consumers (7%, 14/190) and consumers (11%, 28/247; *p* = 0.323), including subgroups based on frequency of consumption in comparison to non-consumers (1–5 times/year: 13%, 19/148 vs. 7%, 14/190; *p* = 0.104; >5 times/year: 9%, 9/99 vs. 7%, 14/190; *p* = 0.677).
  Raw mussel or oyster consumption was also examined. Among participants, 374 reported never consuming raw mussels or oysters (9% anti-HEV IgG positive), while 63 occasionally consumed them (13% anti-HEV IgG positive; *p* = 0.491). Even among those who ate raw products more frequently (≥ 6 times/year: 20%, 2/10), no statistically significant association with increased seroprevalence was observed.
2. Testing commercial Baltic Sea mussels for HEV:
  PCR analysis of 40 commercially sold *Mytilidae* from the Baltic Sea detected no HEV RNA (0%).
3. Experimental HEV exposure in live mussels:
  In the exposure experiFig. (Fig. 2; Table 2), 50 live *Mytilidae* were placed in an aquarium with HEV-contaminated water. HEV RNA was detected in the water sample one hour after exposure. Seven days post-exposure, 10/10 harvested mussels tested positive for HEV RNA in their digestive tracts, with one out of 10 additionally harvested mussels testing positive 14 days post-exposure (Table 2). No HEV RNA was detected in gonads or muscle tissue at any time. By qualitative PCR, no further HEV RNA was detected in any tissue beyond 14 days. Pre-exposure water and mussel samples were all PCR-negative.

**Table 2:**
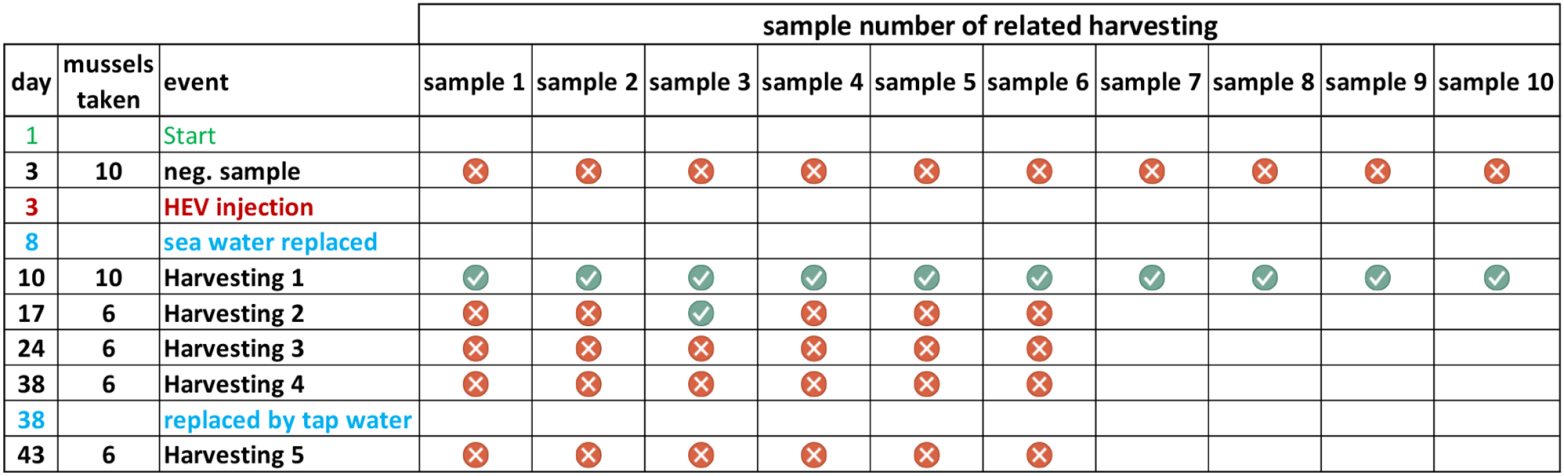
Results of HEV testing of mussel stomach and sequence of events

These findings collectively suggest a minimal risk of HEV transmission from Baltic Sea mussels, even under experimental exposure conditions.

## Discussion

This study provides valuable insights into the potential role of Baltic Sea *Mytilidae* mussels in transmitting hepatitis E virus (HEV). The findings demonstrate that commercially available mussels tested negative for HEV RNA, and experimentally exposed mussels carried the virus transiently in their digestive tract but not in muscle or gonad tissues. Importantly, no evidence of HEV replication was observed, and the virus was cleared from the mussels’ digestive tract by day 24 post-exposure. These results suggest that Baltic Sea mussels pose a minimal risk of HEV transmission to consumers. Furthermore, no significant association was found between mussel consumption and anti-HEV IgG seroprevalence, underscoring the low likelihood of mussels contributing to HEV exposure in Germany.

While these findings are reassuring, the study has limitations that warrant further investigation. One significant limitation is the small sample size of commercially available mussels tested. To entirely exclude the risk of HEV transmission, a larger dataset that includes mussels from different regions of the Baltic Sea and other commercial sources is necessary. Additionally, the ecology and epidemiology of HEV in the Baltic Sea remain poorly understood. Studies exploring HEV contamination pathways, environmental persistence, and interactions with mussel biology would provide a more comprehensive understanding of HEV risks in this region.

Another limitation relates to the experimental conditions. While salt-enriched tap water was necessary due to the unavailability of Baltic Sea water, it may not fully replicate the natural environment, potentially influencing the mussels’ viral clearance dynamics. Furthermore, the precise origins of the supermarket-purchased mussels could not be confirmed. This underscores the need for better traceability of mussel products to identify potential regional variations in HEV prevalence along the German coastline.

Despite these limitations, this study highlights a stark contrast between Baltic Sea mussels and those from other regions, such as Spain and Scotland, where higher rates of HEV positivity have been reported. These findings could inform future risk assessments and contribute to developing regional risk stratification systems for shellfish safety. However, additional research is needed to better understand the biology, ecology, and epidemiology of HEV in mussels, particularly in the context of the Baltic Sea, to ensure robust conclusions about public health risks.

## Data Availability

The data that support the findings of this study are available from the corresponding author upon reasonable request.

## Abbreviations

ELISA: Enzyme-linked immunosorbent assay
HEV: Hepatitis-E-Virus
IgG: Immunoglobulin G
PCR: Polymerase chain reaction
qPCR: Quantitative PCR
RNA: Ribonucleic acid
UV: Ultraviolet
WHO: World Health Organization
MSC: Marine Stewardship Council
